# An end-to-end deep learning architecture for extracting protein–protein interactions affected by genetic mutations

**DOI:** 10.1093/database/bay092

**Published:** 2018-09-18

**Authors:** Tung Tran, Ramakanth Kavuluru

**Affiliations:** 1 Department of Computer Science, University of Kentucky, Lexington, KY, USA; 2 Division of Biomedical Informatics, Department of Internal Medicine, University of Kentucky, Lexington, KY, USA

## Abstract

The BioCreative VI Track IV (mining protein interactions and mutations for precision medicine) challenge was organized in 2017 with the goal of applying biomedical text mining methods to support advancements in precision medicine approaches. As part of the challenge, a new dataset was introduced for the purpose of building a supervised relation extraction model capable of taking a test article and returning a list of interacting protein pairs identified by their Entrez Gene IDs. Specifically, such pairs represent proteins participating in a binary protein–protein interaction relation where the interaction is additionally affected by a genetic mutation—referred to as a PPIm relation. In this study, we explore an end-to-end approach for PPIm relation extraction by deploying a three-component pipeline involving deep learning-based named-entity recognition and relation classification models along with a knowledge-based approach for gene normalization. We propose several recall-focused improvements to our original challenge entry that placed second when matching on Entrez Gene ID (exact matching) and on HomoloGene ID. On exact matching, the improved system achieved new competitive test results of 37.78% micro-F1 with a precision of 38.22% and recall of 37.34% that corresponds to an improvement from the prior best system by approximately three micro-F1 points. When matching on HomoloGene IDs, we report similarly competitive test results at 46.17% micro-F1 with a precision and recall of 46.67 and 45.59%, respectively, corresponding to an improvement of more than eight micro-F1 points over the prior best result. The code for our deep learning system is made publicly available at https://github.com/bionlproc/biocppi_extraction.

## 1 Introduction

Precision medicine is an emerging disease treatment paradigm in which healthcare is customized to each individual patient. To support this effort, it is important to be able to extract useful translational information such as mentions of relationships between genes (given proteins are biochemical materials resulting from expression of corresponding genes, the terms gene and protein are used interchangeably in this paper and the exact meaning is dependent on the context), mutations and diseases. BioCreative (Critical Assessment of Information Extraction in Biology) [12] is an initiative with the aims of providing a standard evaluation framework for assessing text mining systems with respect to relevant problems in the biomedical domain. The related challenges are important as they provide an avenue for introducing new gold standard datasets to the research community that are hand-annotated by human domain experts. The precision medicine track of BioCreative VI, specifically, was organized to identify and study mutations and their effect on molecular interactions. Concretely, this track focuses on mining biomedical literature for protein–protein interactions (PPIs) that are affected by the presence of a genetic mutation. As an example, consider the following sentence: ‘We found that dominant-negative mutants of PML blocked AXIN-induced p53 activation, and that AXIN promotes PML SUMOylation, a modification necessary for PML functions.’ Here, we see that ‘AXIN’ and ‘PML’ are proteins that interact, as indicated by the assertion that ‘AXIN’ promotes SUMOylation in ‘PML’; moreover, a mutation of ‘PML’ is involved. Based on this observation, we can deduce that ‘AXIN’ and ‘PML’ are interesting pairs of proteins to study. We refer to this particular type of relation, where the participants of a PPI are also affected by a mutation, as a PPIm relation. This challenge is important as there has been a lack of tools that allows for the extraction of such interactions from biomedical literature despite its potential to support approaches in precision medicine.

The precision medicine track involves the following two distinct tasks: ‘document triage’ and ‘relation extraction’. In the first task, participants are asked to build systems able to determine whether a PubMed citation is ‘relevant’ or ‘not relevant’ with respect to the relation extraction task; that is, whether or not it contains any PPIm relations to be extracted. In the second task, we are asked to build systems that take as input a PubMed citation and output any PPIm relations along with the Entrez Gene IDs (https://www.ncbi.nlm.nih.gov/gene/) of the participating genes. Thus, for the second task, besides the input text, no additional information is provided making it a true end-to-end requirement where gene spotting, normalization and interaction detection are all required.

In this paper we exclusively focus on the PPIm extraction task and propose a pipeline of the following three modular components: named entity recognition (NER), gene mention normalization (GN) and relation classification (RC). The input to the pipeline is a PubMed article and the output is a set of extracted PPIm pairs. The first component identifies spans of text corresponding to gene mentions. The second component maps the gene mentions to their normalized Entrez Gene IDs. Lastly, the third component classifies all pairs of unique gene IDs found in the article as either positive or negative for the PPIm relation. The system we present here is an improved version of our original challenge entry [35] with three major changes. First, we use GNormPlus [38] to augment the original training corpus with additional gene annotations. For the NER component, this has the effect of reducing mixed signals stemming from the lack of annotations in the original training data. For the RC component, this provides many more meaningful negative examples such that the label imbalance more accurately reflects real-world situations. Second, during testing, we tag sequences of tokens that are missed by the NER component but appear in a gene lexicon (provided with the BioCreative II Gene Normalization training data [26]) to boost overall recall. Third, we consult PubTator [37] as a secondary reference (in addition to the gene database lookup; more later) for document-level gene annotations when mapping genes to their Entrez Gene IDs. We find that these changes drastically improve recall while retaining high precision.

The PPIm extraction task differs from a typical relation extraction task in three notable ways. First, a protein may interact with itself, which implies that a protein can participate simultaneously as both the ‘subject’ and the ‘object’ of a PPIm relation. Second, directionality of a protein pair is immaterial, which implies that $(A,B)$ and $(B,A)$ are equivalent for the sake of system evaluation. Here, the interaction type is also not important as in other PPI tasks so each relation can sufficiently be represented as a pair instead the usual (‘subject’, ‘predicate’, ‘object’) triple. Lastly, it is possible for relations to be expressed across sentence bounds such that the subject and object mentions of a PPIm pair are in different sentences. Hence, we believe it is better to make RC decisions (i.e. extract protein pairs) at the document level for this particular task. This is opposed to sentence-level relation extraction where sentences are assumed to be mutually independent when extracting relations and only pairs mentioned in the same sentence are considered as valid candidates for extraction. Document-level extraction has an additional advantage in that it takes into account sentence-level correlations such as order of sentences expressed.

In the rest of the manuscript, we discuss other approaches to this task and provide an overview of deep neural network architectures in Section 2. We present our main methods in Section 3 and discuss system performance and comparisons in Section 4.

## 2 Background and related work

In this section we cover some basic background on deep neural networks, general prior efforts in biomedical relation extraction and the top performer of the PPIm extraction task we address in this manuscript.

**Deep Neural Networks** Recent progress in natural language processing (NLP) in general has mostly been a consequence of advances in ‘deep’ neural networks—neural networks with at least two layers between the input and output layer and capable of composing useful intermediate representations. Convolutional neural networks (CNNs) in particular were originally developed for image recognition tasks [18] and have been successfully applied to the text domain by exploiting so-called neural word embeddings [17, 33]. These word embeddings represent words as vectors and can be pre-trained using unsupervised methods and further trained when learning on a specific task. CNNs exhibit geometric translational invariance, which allows them to detect contextual features while being insensitive to changes of a translational nature. Using CNNs along with neural word embeddings has been shown to be effective in many natural language tasks (including text classification and relation extraction) since they naturally capture syntactic and semantic information [3, 7, 24].

Recurrent neural networks (RNNs) involving cyclical connections offer another type of neural architecture that has been successfully applied to NLP tasks involving sequence data such as part-of-speech tagging, NER and machine translation [2, 14]. RNNs are a natural architecture for modeling sequences where outputs from previous time steps are fed back as input to the network. It is typical to compose RNNs in both the forward and the backward direction as this allows the sequence to be modeled in both directions in a joint architecture called a bidirectional RNN (Bi-RNN). In a typical Bi-RNN architecture, both the forward and backward RNN receive the same input and are composed independently; once composed, the output vector is typically concatenated at each corresponding time step. Bi-RNNs are important for sequence labeling tasks as the full context is taken into account when assigning a label for each timestep of the input sequence. In this study, we used Bi-RNNs with a more powerful recurrent unit called ‘long short-term memory’ (LSTM) [11, 13] units in the hidden layer that are simply termed Bi-LSTMs.

**Biomedical Relation Extraction** Many early works on relation extraction preprocess the input as a dependency parse tree [4, 29] and exploit features corresponding to the shortest dependency path between candidate entities; this general approach has also been successfully applied in the biomedical domain [1, 10, 20, 32], where they typically involve a graph kernel based Support Vector Machine (SVM) classifier [20, 32]. The concept of network centrality has also been applied [27] such that gene networks were created with respect to a specific disease; genes are then ranked according to network centrality metrics where highly ranked genes were considered more likely to be associated with the disease. Other studies, such as the effort by Frunza *et al*. [9], apply the more traditional ‘bag-of-words’ approach focusing on syntactic and lexical features while exploring a wide variety of classification algorithms including decision trees, SVMs and Naïve Bayes. More recently, innovations in relation extraction have centered around designing meaningful deep learning architectures. Liu *et al*. [21] proposed a dependency-based CNN architecture wherein the convolution is applied over words adjacent according to the shortest path connecting the entities in the dependency tree, rather than words adjacent with respect to the order expressed, to detect drug–drug interactions (DDIs). In Kavuluru *et al*. [16], ensembling of both character-level and word-level RNNs is further proposed for improved performance in DDI extraction. Raj *et al*. [30] proposed a deep learning architecture such that word representations are first processed by a bidirectional RNN layer followed by a standard CNN, with an optional attention mechanism towards the output layer. Luo *et al*. [22] proposed convolving over not only the sentence, but rather over the following five segments of a sentence: before the first entity mention, the first entity mention, in between the entity mentions, the second entity mention and after the second entity mention. A single representation of the candidate relation and its context are then composed via simple concatenation of the CNN outputs. Recent studies have also explored joint modeling of both NER and relation extraction in an end-to-end fashion via deep neural networks [15, 25, 39].

**Top Performing PPIm Extraction Entry** Chen *et al*. [5] produced the best micro-F1 scores during the BioCreative VI PPIm extraction challenge. They used the GNormPlus [38] tool as an ‘out-of-the-box’ solution for recognizing and normalizing gene mentions. The main contribution lies in the RC aspect in which two different approaches are explored. The first is based on a rule-based system using the heuristic that if a protein–protein pair occur together in more than $N$ sentences then it is considered positive for a PPIm relation. This works surprisingly well, which is likely due to the observation that an article that has already been deemed relevant during document triage phase is likely topically-focused on a specific PPIm relation. It is reasonable to assume that two proteins mentioned together multiple times are more likely to be part of a relation than not. They found that $N=2$ was optimal during validation. The second approach is based on traditional SVM with a graph kernel where the input is a dependency graph. Syntactic dependency graphs generated for each sentence are used as classifier features. In case a protein pair is mentioned across two sentences, an artificial root node is generated connecting the roots of the two sentences to form a single larger graph to be used as input. They additionally experimented with introducing handpicked mutation-context binary features in the form of 30 interaction terms including ‘interact’, ‘complex’, ‘bound’, ‘bind’ and ‘regulate’. From the 5-fold cross validation results on the training set, they found that SVM with these mutation features worked best at 27.5% F1. This is contrary to the test results, in which the rule-based approach was superior at 37.67% on the official test set. The authors note an end-to-end performance ceiling of 56% F1 when using GNormPlus for protein recognition and normalization. This aligns with our observation that improving the gene annotation aspect plays a key role in improving overall performance. The system we propose in this paper uses more elaborate heuristics for the NER and gene normalization components and exploits recent advances in deep neural networks for NLP. Our current results improve upon Chen *et al*.’s best results during the challenge by three micro-F1 points in exact matching and by over eight micro-F1 points in homolog-level matching strongly indicating that our end-to-end formulation is more suitable for this task.

## 3 Method

For the relation extraction subtask, we propose a pipeline system that consists of the following three components: supervised NER for gene mention detection, knowledge-based gene normalization and supervised RC to predict each pair of genes found as either positive or negative for an interaction. It is possible to use an ‘out-of-the-box’ solution such as GNormPlus that identifies both gene mentions and their corresponding gene identifier directly; however, we opted for a supervised approach that lets us leverage the generous gene annotations provided with the training corpus for this task. In the rest of this section, we first describe the dataset to be used in Section 3.1. We describe the NER system used to identify spans of text corresponding to a gene mention in Section 3.2. We then describe our knowledge-based method for gene normalization in Section 3.3 and RC model in Section 3.4.

### 3.1 PPIm Dataset

The PPIm dataset consists of 597 article titles and abstracts each of which is annotated with gene mentions and interacting relevant protein pairs (at least one per citation) identified by their Entrez Gene IDs. In total, there are 752 pairs such that each article contains 1.26 relevant PPIm pairs ‘on average’. It is important to note that a gene is only annotated with mention-level offsets if it exists as part of a PPIm relation in the ground truth; hence, these gene annotations are incomplete for the sole purpose of training an NER model to identify gene mentions. The test has 632 articles each with at least one PPIm pair and a total of 868 PPIm pairs over the full test set; here we observe a similar distribution to the training set with an average of 1.37 pairs per article. Systems designed for this task are officially evaluated using standard metrics such as micro and macro F1/precision/recall; additionally, evaluations can be performed using exact or homologous gene matching. Further details of system evaluation are discussed in Section 4.

### 3.2 Gene mention identification (NER)

**Figure 1:**
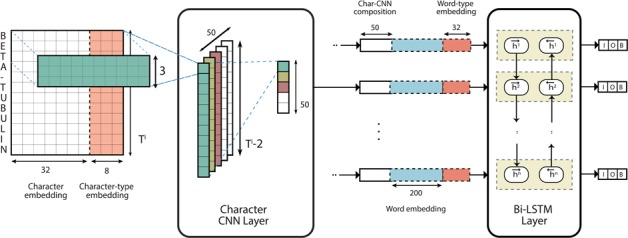
Deep neural network architecture of the NER model.

The aim of the first component in the pipeline is to identify spans of text corresponding to gene mentions. To that end, we propose the use of a deep neural network system based on a CNN–LSTM hybrid model initially proposed by Chiu *et al*. [6] for NER. This sequence-to-sequence model composes word representations with CNNs by convolving over character *n* grams. At the word level, contextual word representations are composed using a bi-directional LSTM layer. A separate fully-connected softmax output layer is present at the output of each LSTM unit such that an Inside–Outside–Beginning (IOB; The IOB format is a tagging scheme commonly used in NER and sequence labeling tasks. The Inside and Beginning tags indicate that the tag is inside and at the beginning of a typed span, respectively, while Outside indicates that the tag is outside of a span. Typically, and in our model, the Beginning tag is only used when a tag is followed by a tag of the same type to indicate the start of a new span.) [31] label prediction can be made for each token. A visualization of the architecture can be observed in Figure 1.

Herein, we formulate the model from the bottom up. In this formulation, a word at position $i$ for $i = 1, \ldots , n$ is treated as a lowercased character sequence $c^{i}_1, \ldots , c^{i}_{T^i}$ represented by their index into the character vocabulary $\mathcal{V}^{\textrm{char}}$. The corresponding character embedding matrix $E^{\textrm{char}} \in{\mathbb{R}}^{|\mathcal{V}^{\textrm{char}}| \times \alpha }$ embeds each character as a vector of length $\alpha $ (a hyperparameter). Embedding matrices can be initialized to random or pretrained values; in either case, the word vectors are (further) modified via backward propagation. We use the same embedding set-up to produce ‘character type’ embedding vectors of length 8 indicating the type of character: lowercase, uppercase, punctuation or other. Suppose the embedding matrix for ‘character type’ is $E^{\textrm{ctype}} \in{\mathbb{R}}^{4 \times 8}$ and $z^{i}_1, \ldots , z^{i}_{T^i}$ represents the sequence of enumerated character types for the word at position $i$. The word at position $i$ can then be represented as a matrix composition $B^i$ of its character embeddings, or concretely

\begin{equation*} B^i = \left(\!\! \begin{array}{c} E_{[c_{i}^{1}]}^{\textrm{char}} {||} E_{[z_{i}^{1}]}^{\textrm{ctype}} \\ \vdots \\ E_{[c_{{i}^{i}}^{T}]}^{\textrm{ctype}} {||} E_{[z_{{i}^{i}}^{T}]}^{\textrm{ctype}} \\ \end{array} \!\!\right), \end{equation*}

where $ E_{[j]}^{\textrm{char}} $, $ E_{[j]}^{\textrm{ctype}} $ is the ${j}{\textrm{th}}$ row of $E^{\textrm{char}}$, $E^{\textrm{ctype}}$, respectively and $\mathbin{\|}$ is the vector concatenation operator. The central idea in CNNs is the so-called ‘convolution’ operation over the document matrix (or in this case, the ‘word’ matrix) to produce a feature map representation using a ‘convolution filter’ (CF). The convolution operation * is formally defined as the sum of the element-wise products of two matrices. That is, for two matrices A and B of same dimensions, $A * B = \sum _{j} \sum _{k} A_{j,k} \cdot B_{j,k}$. We perform a convolution operation over $B^i$ of window size 3 to obtain the feature map $\mathbf{v}^i = [v^i_1, \ldots , v^i_{T^i-2}]$ such that

\begin{equation*} v^i_j = \textrm{ReLU}(W^{\textrm{char}} * [i]{B}{j}{j+2} + b^{\textrm{char}}), \end{equation*}

where $ [i]{B}{j}{j+2}$ is a window of matrix $B^i$ spanning from row $j$ to row $j+2$, $W^{\textrm{char}}$ and $b^{\textrm{char}}$ are network parameters representing a CF, and the linear rectifier activation function $\textrm{ReLU}(x) = \max (0,x)$. The goal is to learn multiple CFs that can collectively capture diverse representations of the same word. Here, specifically, we learn $\kappa $ filters to obtain $\kappa $ corresponding feature maps denoted as $\mathbf{v}^{i,1}, \ldots , \mathbf{v}^{i,\kappa }$. As a crucial step with CNNs, we select the most distinctive feature of each feature map using a max-over-time pooling operation [8]. Let $\mathbf{v}^{i,j}_k$ be the ${k}{\textrm{th}}$ value of $\mathbf{v}^{i,j}$, then the word representation at position $i$ is $\mathbf{u}^i = [\hat{v}^{i,1}, \ldots , \hat{v}^{i,\kappa }],$ where $\hat{v}^{i,j} = \max \, ( \mathbf{v}^{i,j}_{1}, \ldots , \mathbf{v}^{i,j}_{T^i-2})$. Conceptually, we can roughly equate this to composing a word representation using the traditional ‘bag-of-words’ model, except here the features consist of character tri-grams. Because of the way max-pooling is applied, the order of tri-grams is immaterial.

Once a representation is composed for each word, we then use a bi-directional LSTM to model the word sequence. It is important that we also include actual word embeddings (in addition to those obtained through character embedding compositions) as well as ‘word-type’ embeddings as input. The latter embeddings serve a similar purpose to that of the ‘character types’ and can correspond to one of the five following types: all lowercase, mixed-cased, capitalized first letter, all uppercase or other. We now transition to a word-level perspective. Formally, the input to the network is a sequence of word indexes $w_1, \ldots , w_{n}$ into the word vocabulary $\mathcal{V}^{\textrm{word}}$ and the corresponding embedding matrix is denoted as $E^{\textrm{word}} \in{\mathbb{R}}^{|\mathcal{V}^{\textrm{word}}| \times d}$. In addition, we denote $\bar{z}_1, \ldots , \bar{z}_{n}$ as a sequence of enumerated ‘word types’ corresponding to the embedding matrix $E^{\textrm{wtype}} \in{\mathbb{R}}^{5 \times \alpha }$. The bi-directional LSTM with a hidden/output unit size of $\pi $ can then be composed as

\begin{align*} \overrightarrow{\mathbf{h}}^i &= \textrm{LSTM}^{\rightarrow}\left( \mathbf{u}^i \big{\|} E_{[w_{i}]}^{\textrm{word}} \big{\|} E_{[\bar{z}_{i}]}^{\textrm{wtype}}, \, \overrightarrow{\mathbf{h}}^{i-1} \right),\\ \overleftarrow{\mathbf{h}}^i &= \textrm{LSTM}^{\leftarrow}\left(\mathbf{u}^i \big{\|} E_{[w_{i}]}^{\textrm{word}} \big{\|} E_{[\bar{z}_{i}]}^{\textrm{wtype}}, \, \overleftarrow{\mathbf{h}}^{i+1} \right),\\ \mathbf{h}^i &= \overrightarrow{\mathbf{h}}^i {\|} \overleftarrow{\mathbf{h}}^i\qquad \textrm{ for } i = 1,\ldots,n, \end{align*}

where $\mathbf{u}^i$ is character based embedding for $w_i$, $E^{\textrm{word}}_{[j]}$ and $E^{\textrm{wtype}}_{[j]}$ are ${j}{\textrm{th}}$ rows of $E^{\textrm{word}}$ and $E^{\textrm{wtype}}$, respectively, and $\textrm{LSTM}^{\rightarrow }$ and $\textrm{LSTM}^{\leftarrow }$ represent an LSTM unit composition in the forward and backward directions, respectively. The concatenated output vector $\mathbf{h}^i \in{\mathbb{R}}^{2\pi }$ represents the entire context centered at the ${i}{\textrm{th}}$ word. The output at each timestep necessarily has its own softmax output layer in order for the network to be able to tag each word with an IOB label typically used for NER. The output at each position $i=1,\ldots , n$ is

\begin{equation*} \mathbf{q}^i = W^{out} \mathbf{h}^i + b^{out}, \end{equation*}

where $W^{out} \in{\mathbb{R}}^{m \times 2\pi }$ and $b^{out} \in{\mathbb{R}}^{m}$ are network parameters and $m=3$, the number of NER tags (‘B-GENE’, ‘I-GENE’ and ‘O’). In order to get a categorical distribution, we apply the softmax formulation to $\mathbf{q}^i$ such that

\begin{equation*} \mathbf{p}^i_j = \frac{e^{\mathbf{q}^i_j}}{\sum_{l=1}^{m}{e^{\mathbf{q}^i_l}}}, \end{equation*}

where $\mathbf{p}^i$ is the vector of probability estimates serving as a categorical distribution over gene IOB tags for the word at position $i$. We optimize by computing the standard categorical cross-entropy loss at each output layer. Since each instance may be of a different sequence length, the final loss is computed as the ‘mean’ over all $n$ losses, one per word. The per-example loss $\ell $ is therefore computed as

\begin{equation*} \ell = - \frac{1}{n} \sum_{i=1}^{n}\sum_{j=1}^{m} \mathbf{y}^i_j \log\left( \mathbf{p}^i_j \right), \end{equation*}

where $\mathbf{y}^i \in{\mathbb{R}}^m$ is the correct label for word $i$ encoded as a one-hot vector. Next, we discuss the training procedure and model configuration.

**Training and Model Configuration** The NER model is trained on the training data and additionally on the GNormPlus corpus that includes re-annotations of the BioCreative II GM/GN corpus [26]. The core training data consists of 5668 sentence-level training examples while the GNormPlus corpus constitutes an additional 6389. We chose an embedding size of $\alpha =32$ with $\kappa =50$ filters for the character-based CNN composition. These hyperparameters were chosen based on the results of a hyperparameter search conducted by Chiu *et al*. [6] and further tweaked during initial experiments. At the word level, we used word embedding vectors of size $d=200$ pre-trained on the PubMed corpus [28]. The forward and backward LSTM are each implemented with a hidden unit size of $\pi = 200$. The network was trained using Stochastic Gradient Descent (SGD) with an exponential decay rate of 0.95 for a maximum of 10 000 iterations. On each iteration, we trained the network using a mini-batch [19] of 20 random examples. We checkpointed every 100 iterations and saved only the checkpoint with the best F1 on the development set. We also deployed ‘early stopping’ such that training is stopped if there are no improvements for 10 checkpoints. We train 10 such models (each with a different seed) as part of an ensemble where each model is trained on a smaller random subset of only 50% of the original training set. We observed that the ensemble was less prone to overfitting (during initial experiments) when each model of the ensemble was only exposed to a smaller subset of the training data.

**Augmented Gene Annotations** An issue with the gene annotations in the training data is that they are not comprehensive. In fact, only genes participating in at least one relationship are annotated with mention-level offsets and gene IDs. This issue manifests in the following two distinct ways:

1. Mixed signals are introduced during learning (for the NER model) given it is possible for the same entity to appear as a target (annotated with ‘I-GENE’) for identification in one training example but not others (they are instead annotated with *O*) where it may not participate in an interaction. Due to the nature of a pipeline system, downstream bottlenecks can often occur as a result of low recall at the front-end of a pipeline. If we fail to identify a gene mention, for example, we will miss any relations it may participate in regardless of the competency of the RC component.
2. Data generated to train the RC component will not contain enough meaningful negative examples given gene mentions in the original training dataset are limited to those participating in interactions. From a manual observation of the data, we find that most examples generated are positive with many of the negative instances resulting from self-interactions.

From our original system submission [35], we found that models trained on only the provided annotations worked reasonably well despite the highlighted issues. As a strategy to overcome these issues and to improve end-to-end recall, we augment gene annotations provided in the training set using the PubTator tool [37] (which uses GNormPlus [38] as the backend for gene annotations). We simply run PubTator on the training corpus and insert genes it finds to corresponding spans of text in the training data that have consecutive ‘O’ labels. The augmented corpus is instead used for training the supervised model (not only for NER, but RC as well). When doing this, we make sure to apply corrections such that the label sequence conforms to IOB rules.

**Post-processing step** Before proceeding to the gene normalization component, we perform a post-processing step to the output of the NER system in an attempt to maximize recall. Specifically, we use the gene lexicon provided with the BioCreative II Gene Normalization training data [26] as a knowledge source. The gene lexicon provides mappings of gene mentions to potential Entrez Gene IDs (keeping in mind that a gene mention may map to more than one unique ID). For a document input, we search for occurrences of gene mentions from the lexicon (note that we prioritize longer gene mentions over shorter ones) and add them as additional mentions to our supervised NER system’s annotations barring those that overlap with our NER gene spans. In the gene normalization step (to be discussed next), we filter out gene mentions for which there are no plausible gene ID mappings. As such, the lower precision at the NER level due to this recall oriented post-processing step is reconcilable as we can weed out obviously bad gene mentions during gene normalization; hence, precision can be compromised for the sake of improved recall for the NER component.

### 3.3 Entrez Gene ID Normalization (GN)

For the gene normalization component, we initially experimented with a naive look-up approach using the gene lexicon from BioCreative II normalization task [26] as well as mappings provided with the training corpus. This served as a reasonable baseline; however, it does not take context into consideration during the mapping process. A gene mention may be incorrectly mapped to one of its many homologs resulting in increased false positives. The final version of our gene normalization system is knowledge-based and more sophisticated in that it takes into consideration both the gene mention and the context. This system relies on the National Center for Biotechnology Information (NCBI) gene database [23] to identify the candidate gene IDs for a particular mention and further narrows it down to a ‘best guess’ based on the document in which it occurred. We define two utility functions that serve as the basis for this system. Before we proceed, we recall that the full citation (title and abstract) represents a single input instance for our task. Hence, the context for confirming the mapping is the Medline citation of the full article.

The first function, ‘gene_name_lookup’, takes as input a mention span and returns a list of candidate gene IDs sorted by relevance. This is achieved by querying the NCBI gene database via the E-utilities Application Programming Interface (API) (an example query for the gene span ‘Utp21’: https://eutils.ncbi.nlm.nih.gov/entrez/eutils/esearch.fcgi?db=gene&term=Utp21&retmax=100&sort=relevance). This provides a ranked list of candidate genes for a given gene mention.

The intuition here is that the top few in this list are either the correct gene or at least homologs of the correct gene. We now define the second function, ‘gene_pmid_lookup’, which takes as input a PubMed article ID (PMID) and returns a list of candidate gene IDs for the article. We achieve this by making another query to the NCBI gene database using the PMID of the current document as query input (an example query for the PMID 18725399: https://eutils.ncbi.nlm.nih.gov/entrez/eutils/esearch.fcgi?db=gene&term=18725399[PMID]). This allows us to narrow down the list of candidate gene IDs to ones that have already been identified as appearing in the document.

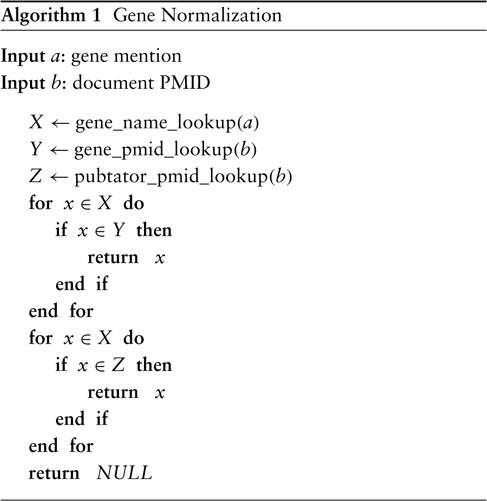

The final ‘gene_normalization’ algorithm takes as input a gene mention and a PMID and returns either a gene ID or ‘NULL’. The latter indicates that no match can be found, in which case we simply ignore the span entirely for the remainder of the pipeline. From initial experiments, we found that relying only on the NCBI gene database to inform us of the possible genes for a document is too limiting and hurts recall considerably. This is because, while it is very precise, the database is not a comprehensive source of knowledge (at least for our purpose) and should not be relied upon as such. Hence, there is reason to believe that augmenting it with another high-precision system such as PubTator would improve overall recall. Let ‘pubtator_pmid_lookup’ be a function that takes as input a PMID and returns a list of candidate genes for an article—not unlike ‘gene_pmid_lookup’. The difference is that ‘pubtator_pmid_lookup’ returns the output of PubTator for the article without any information about word-level offsets; in other words, only a list of document-level gene IDs is returned. A natural union works well in our experiments, but we find a slight advantage in using ‘gene_pmid_lookup’ as the primary source of knowledge with ‘pubtator_pmid_lookup’ serving as a secondary fallback. The final version of the procedure is defined in Algorithm 1.

### 3.4 RC of Gene Pairs (RC)

**Figure 2:**
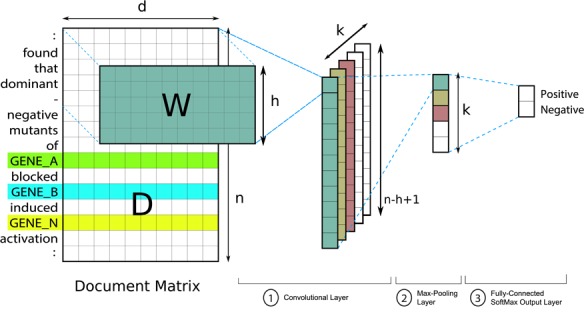
Network architecture of the RC model (adapted from Tran and Kavuluru [36, Figure 1]).

To extract PPIm pairs, we propose using a deep neural network architecture based on CNNs for RC. The proposed model was originally introduced by Kim *et al*. [17] for text classification and later adapted by us for narrative-based prediction of mental conditions [36]. An overview of the architecture modified to suit the RC task is presented in Figure 2. Since the architecture is identical (with exception of the output layer) to our prior work [36], we simply refer readers to the original study for the exact model formulation; the remainder of this section will instead focus on the training and configuration aspect of the model.

**Training and Model Configuration** When generating training examples for this model, we use the ‘augmented’ training corpus as described in Section 3.2 with the additional gene mentions. For this task, each pair of candidate genes in an article constitutes a separate candidate interaction. Hence, for each pair of candidate genes, we generate a distinct training instance by performing well-known entity-binding—we replace mentions of the pair with special tokens GENE_A and GENE_B (with their own embeddings) in the corresponding document text. We adapt this idea of entity-binding from prior efforts [16, 21] on classifying DDIs, which obtained competitive results on a popular DDI dataset. For a gene pair $(A,B)$, we also generate an additional instance for the reverse case $(B,A)$ given directionality does not matter. Note that we run both cases of a candidate pair during testing and take the average output score for classification. We also generate examples for the exception case when the candidate pair involves the same gene, i.e. $A = B$, in which case GENE_S is used for entity binding of the single gene ID. We also replace mentions of other genes in the narrative with a special GENE_N token in either case. In total, we generated 2972 instances from the 597 articles in the training set. At test time, we only predict pairs as positive where the mean probability is above 50% for the instance generated from $(A,B)$ and its reverse case $(B,A)$. In case no pairs meet the threshold, we make a single positive prediction by choosing the pair with the highest probability (even if it is $\leq \!50\%$).

We now describe the configuration of the RC model. As with the NER model, we used word embeddings of size 200 pre-trained on the PubMed corpus [28]. For the convolutional component, we used window sizes of 3, 4 and 5 with 200 convolutional filters. The model was trained for 30 epochs using RMSProp [34] (an SGD variant) using mini batches [19] with a batch size of 8 and a learning rate of 0.001. Since each instance is a collection of sentences and the window size is at most 5, we pad four zero-vectors at the beginning and the end of the input text as well as between sentences. We additionally apply dropout at a rate of 50%. During training, we checkpoint model parameters at each epoch and only keep the checkpoint resulting in the highest F1 on the development set. We train 10 such models as part of an ensemble. Each model of the ensemble is trained and tuned on a random split of 80 to 20% and seeded with a different value for random parameter initialization. The neural network was configured based on insights from our prior work [36] with this particular architecture and further tuned during initial experiments.

## 4 Results and discussion

Officially, systems submitted for this task are evaluated on micro-F1 with macro-F1 being a secondary metric introduced after the original challenge. There are two matching criteria that are considered when evaluating: exact gene ID matching and HomoloGene Gene ID matching. In the latter case, genes of the same homology group are considered equivalent for the purpose of evaluation. This allows room for errors in the gene mapping aspect of the system and is therefore a less stringent measure compared to ‘exact matches’. To identify homologous genes, the NCBI HomoloGene (https://www.ncbi.nlm.nih.gov/homologene) database is used as a reference. In this context, the macro-F1 metric is based on computing the example-based F1 for each test article and averaging it over all test articles; this is different from the standard macro-F1 in a multi-class setting where it is the average of the F1 score over all classes.

The end-to-end performance of our system on the official test set is recorded in Table 1. Results of the top-performing participants of the original challenge are displayed in order of ascending micro-F1. Our original system submission [35] during the challenge placed second on exact matching (Table 1, row 2) and on HomoloGene ID matching (Table 1, row 6) at a micro-F1 of 30.03 and 37.27%, respectively. As observed in Table 1, we were able to improve drastically on previous results by at least 7 points in micro-F1 for both exact and HomoloGene ID matching. The gains are almost entirely due to the improved recall of the new system although minor gains in precision were also observed. We also included the results of Chen *et al*. [5] for comparison as their system placed first on both matching criteria. Our improved system attains competitive test results for this dataset at 37.78% micro-F1 on exact matching and 46.17% micro-F1 on HomoloGene ID matching.

**Table 1:** System performance on the official test set

	HomoloGene ID Matching	Method	Micro-P (%)	Micro-R (%)	Micro-F (%)	Macro-P (%)	Macro-R (%)	Macro-F (%)
1	✘	Task baseline	10.91	47.41	17.74	19.29	47.16	23.21
2		Tran and Kavuluru [35]	37.39	25.09	30.03	26.86	27.35	25.87
3		Chen *et al*. [5]	40.00	30.84	34.83	28.68	33.53	28.90
4		Our system	38.22	37.34	**37.78**	39.68	40.94	38.46
5	$ \mathbf{\checkmark} $	Task baseline	14.68	51.97	22.90	21.36	51.57	26.02
6		Tran and Kavuluru [35]	46.53	31.09	37.27	32.87	34.15	31.94
7		Chen *et al*. [5]	43.18	33.41	37.67	30.87	35.86	31.09
8		Our system	46.67	45.69	**46.17**	48.53	49.94	47.03

In Table 2, we study the iterative gains achieved by incrementally applying proposed changes to our original system [35]. In order to draw conclusions based on statistical significance, we apply the following experiment. First, we train a set of 30 models each with randomly initialized weights for both the NER and the RC component. Recall that both components make predictions based on ten-model ensembles. We repeatedly evaluate the end-to-end system on the test set 30 times; each evaluation run involves a different ten-model ensemble for each component sampled from their respective pool of 30 trained models. We record the mean-F1 and 95% confidence intervals from these experiments in Table 2. Based on the results of this experiment, we can conclude that performance gains from the proposed changes are statistically significant (with exception of the retrained NER/RC component on HomoloGene ID matching). Next, we discuss these changes in detail.

**Table 2:** Iterative component-level analysis on the official test set

	HomoloGene ID Matching	Method	Micro-P (%)	Micro-R (%)	Micro-F (%)
1	✘	Our base system [35]	35.115 $\pm $ 0.488	25.380 $\pm $ 0.551	29.461 $\pm $ 0.540
2		+ Retrained NER/RC	40.848 $\pm $ 0.148	24.211 $\pm $ 0.094	30.403 $\pm $ 0.112
3		+ Improved NER	42.368 $\pm $ 0.126	25.210 $\pm $ 0.079	31.611 $\pm $ 0.090
4		+ Improved GN	37.425 $\pm $ 0.303	37.221 $\pm $ 0.205	**37.317** $\pm $ 0.194
5		Lexicon-based GN + Our NER/RC	12.149 $\pm $ 0.156	13.826 $\pm $ 0.132	12.925 $\pm $ 0.106
6		GNormPlus-based NER/GN + Our RC	37.069 $\pm $ 0.206	35.637 $\pm $ 0.176	36.333 $\pm $ 0.082
7	$ \mathbf{\checkmark} $	Our base system [35]	44.335 $\pm $ 0.684	31.871 $\pm $ 0.708	37.077 $\pm $ 0.713
8		+ Retrained NER/RC	50.406 $\pm $ 0.161	29.991 $\pm $ 0.092	37.543 $\pm $ 0.113
9		+ Improved NER	52.393 $\pm $ 0.139	31.186 $\pm $ 0.081	39.099 $\pm $ 0.094
10		+ Improved GN	45.989 $\pm $ 0.365	45.863 $\pm $ 0.278	**45.927** $\pm $ 0.251
11		Lexicon-based GN + Our NER/RC	13.592 $\pm $ 0.183	15.594 $\pm $ 0.141	14.517 $\pm $ 0.121
12		GNormPlus-based NER/GN + Our RC	40.067 $\pm $ 0.178	38.632 $\pm $ 0.231	39.329 $\pm $ 0.095

We start by implementing changes to the NER and RC components such that they are trained on the augmented training corpus (recall that this corpus includes the original gene annotations as well as genes identified by GNormPlus). This results in fewer mixed signals for NER component while supplying the RC component with meaningful negative examples. From this, we see a notable improvement in micro-precision of at least 5 points on exact matching and 6 points on HomoloGene ID matching at a minor cost of recall in either case (rows 2 and 8 of Table 2); due to the nature of harmonic means and the fact that the performance already skews toward precision, improvements to micro-F1 are marginal. Next, we change the NER component by adding an NER post-processor that takes the output of the NER component and annotates unmatched gene names using the gene lexicon as a dictionary. From this we observe minor improvements (rows 3 and 9 of Table 2) to both precision and recall corresponding to an increase of at least one micro-F1 that is consistent for either matching criteria. A suspected bottleneck of our system is that it has an overly strict gene mapping criterion in that only genes that are annotated in the NCBI gene database for a particular PMID are allowed. The system is precise, but does not comprehensively cover all genes at the document level. Hence, we implemented a final change such that document-level PubTator (GNormPlus) annotations are used as a secondary recourse when considering the scope of genes to allow for a particular article. This final change is responsible for the most dramatic improvement (rows 4 and 10 of Table 2) to micro-recall at 12 points on exact matching and 14 points on HomoloGene ID matching. This comes with a cost to micro-precision at 5 points on exact matching and 6 points on HomoloGene ID matching. We arrive at relatively balanced precision and recall measures, an observation that is consistent on either matching criteria, resulting in an increase of at about 6 points on exact matching and 7 points on HomoloGene ID matching with respect to micro-F1.

We additionally include results based on other variants of our system for comparison. For example, we report performance for a variant in which the NER and RC component are fixed while replacing the GN component with a method based on gene lexicon mapping and a fuzzy string matching that allowed genes to be mapped to gene IDs within a 90% similarity threshold. This corresponds to rows 5 and 11 of Table 2 in which we observe very poor performance. For HomoloGene ID matching, the result is worse than the baseline reported in Table 1. This is expected as article context is not used to infer the correct gene ID from many possible gene IDs that are homologous in nature. On the other hand, using GNormPlus with the retrained RC component results in surprisingly high performance. This is contrary to our initial experiments on a held-out validation set wherein GNormPlus performs much worse at a micro-F1 of 26.75%—granted this was prior to system improvements as described in this study. This could be an indicator that GNormPlus is better at annotating genes on the test set than the training set. Nevertheless, relying on GNormPlus as the core NER and GN component would result in 36.33 and 39.33% micro-F1 scores on exact and homologous matching, respectively (rows 6 and 12 of Table 2); while these scores are high, this restricts any further improvement to strictly the RC component and the pipeline wide improvements achieved by our system are still superior (rows 4 and 10 of Table 2).

**Figure 3:**
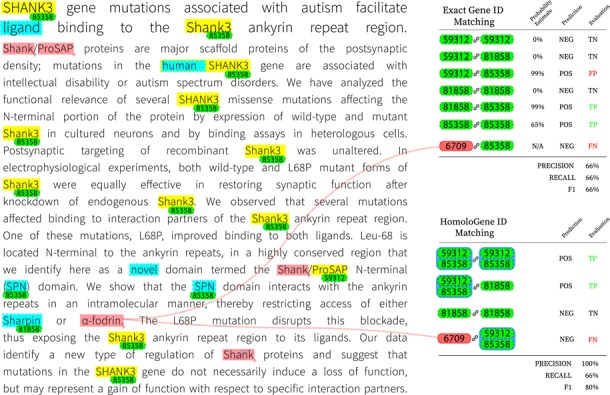
Visualization of decisions made by the final system on article with PMID 23897824.

To gain further insight on the inner workings of the final system, we provide a visualization of intermediate decisions made on a concrete example in Figure 3. The target article, identified by PMID 23897824, was manually chosen from the set of test examples based on its potential for discussion as well as practical considerations (such as length). Highlighted in yellow are spans of text initially identified by the NER system; further corrections to these annotations by consulting the gene lexicon are highlighted in blue. Gene ID annotations are tagged (in green) for each named entity span for which the gene normalization component finds a suitable match. The color red is reserved for spans and genes that were missed entirely by the system. We also include an example-based evaluation on both matching criteria for the final prediction. For HomoloGene ID matching, we group genes that are homologous accordingly.

One clear observation to be made is that most occurrences of the gene *Shank3* are captured by the supervised NER system. Since *Shank3* and its variants do not occur in the training set, this example demonstrates the ability of the system to generalize to unseen examples. Occurrences of the same gene without the numeric suffix are not captured however, which can be an indication that the character-level composition plays an influential role and that there is bias for word tokens that are a mix of alphabetic and numeric characters. We can also observe that the NER component was unable to detect the gene *$\alpha $-fodrin*, more commonly known as *SPTAN1*. This is due to the system’s lack of support for non-ASCII characters; here, we believe a simple preprocessing step to convert non-ASCII characters to a more processable form prior to training and testing will alleviate such issues. The final evaluation of this example shows that missing such genes can be detrimental to overall recall. The post-NER correction step introduces its share of false positives including ‘ligand’ and ‘novel’; nonetheless, it is responsible for detecting the only mention of the gene *Sharpin*, which is a participant of a PPIm relation according to the ground truth. The result is a net gain as the false positives introduced are not normalized by the gene normalization component at this stage and are therefore ignored for the rest of the pipeline. Another observation is that *Shank/ProSAP* individually refer to protein names but in this context may refer to a group of proteins; the first instance of this mention is ignored while the NER system detects only ‘ProSAP’ in the second mention. In this case, ‘ProSAP’ appears to be a source of error as it is ultimately mapped to gene ID 59312, which is *Shank3* but of the variety that occurs in the Norwegian rat. This is in contrast to other instances of *Shank3* that are correctly identified as of the human variety (gene ID 85358). Despite genes 59312 and 85358 being homologous, incorrectly identifying the precise gene ID predictably results in a false positive when evaluating on exact matches. This issue disappears when matching on HomoloGene IDs, as shown in the right panel of Figure 3. To bridge the gap between exact and homologous gene ID matching performance, one option to reduce false positives is by consolidating the gene ID mappings for subsets of unique gene IDs that are homologous; for example, the use of a voting mechanism for deciding the correct variant for all members of the subset. However, it is necessary to consider the trade-off since such a system would not perform well on articles without narrow focus on any particular species of animal.

## Conclusion

In this paper, we proposed an end-to-end deep learning system that consists of NER, gene normalization, and RC for the BioCreative VI Precision Medicine track’s task on relation extraction. We proposed changes to our original system entry for the challenge and analysed the incremental performance gains of these changes. Furthermore, we demonstrated that the proposed system performs competitively for this task by significantly improving upon top results achieved in the original challenge. We believe this is an important progression in supporting efforts in precision medicine. A drawback of the system is the lack of built-in mechanisms for interpretability of decisions, which can be rectified by adding an attention layer to highlight contextual words or phrases that are central to this new problem domain. On the other hand, the lack of comprehensive gene annotations also poses a non-trivial challenge when attempting to build an end-to-end system for this task. The system as proposed relies heavily on numerous external tools and knowledge bases to circumvent the lack of comprehensive gene annotations. As human-expert annotations are expensive and time consuming, this aspect may continue to surface in future datasets of a similar nature. Our future efforts will focus on dealing with this aspect in a more direct fashion while realizing a true end-to-end deep neural network that is able to model all components jointly.
